# Evaluation of the Relative Performance of the Subflattenings Method for Phylogenetic Inference

**DOI:** 10.1007/s11538-023-01120-z

**Published:** 2023-01-30

**Authors:** Joshua Stevenson, Barbara Holland, Michael Charleston, Jeremy Sumner

**Affiliations:** School of Natural Sciences (Discipline of Mathematics), University of Tasmania, Hobart, Australia

**Keywords:** Phylogenetics, Flattening, Subflattening, Splits, Rank

## Abstract

The algebraic properties of *flattenings* and *subflattenings* provide direct methods for identifying edges in the true phylogeny—and by extension the complete tree—using pattern counts from a sequence alignment. The relatively small number of possible internal edges among a set of taxa (compared to the number of binary trees) makes these methods attractive; however, more could be done to evaluate their effectiveness for inferring phylogenetic trees. This is the case particularly for subflattenings, and the work we present here makes progress in this area. We introduce software for constructing and evaluating subflattenings for splits, utilising a number of methods to make computing subflattenings more tractable. We then present the results of simulations we have performed in order to compare the effectiveness of subflattenings to that of flattenings in terms of split score distributions, and susceptibility to possible biases. We find that subflattenings perform similarly to flattenings in terms of the distribution of split scores on the trees we examined, but may be less affected by bias arising from both split size/balance and long branch attraction. These insights are useful for developing effective algorithms to utilise these tools for the purpose of inferring phylogenetic trees.

## Introduction

The accurate inference of species trees from aligned sequence data is a key task that underpins much of evolutionary biology. However, it is a very challenging statistical problem. Much of this difficulty is because sequence evolution is a highly heterogeneous process. The substitution process can vary in different parts of the evolutionary tree and also in different parts of the genome. In addition to this, the local gene tree along which sequences evolve may vary across the alignment.

There have been two broad approaches in trying to cope with this complexity. The first approach is to model it; likelihood-based and Bayesian approaches have become increasingly parameter rich as they use partitions and/or mixtures to account for heterogeneity. The second approach has been to develop methods that are robust to this kind of complexity and avoid having to estimate what are essentially large numbers of nuisance parameters. For example, the *logDet* method (see Lockhart et al. 1994) allows for consistent inference under the general Markov model (GMM) without having to fit any parameters. Methods based on *phylogenetic invariants* also fall into this class of approaches. While invariant methods were not initially competitive compared to maximum likelihood (Huelsenbeck and Hillis 1993), there has been a renaissance in their use over the last decade or so. Explicit use of algebraic methods in phylogenetics started in the late 1980s with both the *Hadamard conjugation* ideas of Hendy and Penny (1989) and the discovery of *phylogenetic invariants* by Cavender and Felsenstein (1987) and Lake (1987). These initial ideas exploited the underlying symmetries inherent in both the relevant molecular substitution models, e.g., the Kimura 3ST model (Kimura 1981), and the phylogenetic tree itself (leaf permutation symmetries). Importantly, the theory of phylogenetic invariants eventually led to the comprehensive application of methods from algebraic geometry, beginning with the work of Allman and Rhodes (2004, 2007). A breakthrough paper by Allman and Rhodes in 2008 described a new class of invariants based on the idea of a (tensor) *flattening* and the rank properties of matrices of site pattern probabilities.

The *flattening* construction is quite natural to phylogenetics as it corresponds to identifying evolutionary splits (partitions of the set of taxa into two parts, akin to an edge in a tree) by reinterpreting a multiple sequence alignment to consist of two sequences of expanded characters (one sequence for each side of the split). For a split *A*|*B* (on a DNA alignment of $t=|A|+|B|$ sequences), the sequence alignment can be summarised into a $4^{|A|}\times 4^{|B|}$ *matrix* with entry *I*, *J* being the count of alignment locations with subpattern *I* on the left side of the split and subpattern *J* on the right. The biological intuition: if one takes a split corresponding to an edge in the true tree, the probability mass contained in the flattening matrix will be concentrated on certain off-diagonal entries, whereas for true splits, the probability mass will be focused on the diagonal. This information can then be detected by measuring the (approximate) algebraic rank of the flattening matrix, which, in particular, is typically done using the singular value decomposition (Sect. 2). Thus, the flattening matrices provide a very direct means of detecting phylogenetic signal and identifying edges in the tree.

Subsequent papers by Chifman and Kubatko (2015) and Gaither and Kubatko (2016) observed the flattening rank properties in practice, using sequence data generated under the multi-species coalescent and with a distribution of rates across sites.

Using a different perspective and starting point, the work by Sumner and Jarvis (2005); Sumner et al. (2008b) on *Markov invariants* has also taken an algebraic approach to phylogenetics but has instead concentrated on the time evolution of the underlying Markov process. In particular, Sumner and Jarvis (2005) detail a particular set of Markov invariants, the *squangles*. These invariants are simply polynomials in the site-pattern probabilities, which have distinct expected values for each of the three quartet trees, assuming the trees have evolved under the general Markov model. This allows them to be used for quartet reconstruction. We use squangles as a point of comparison alongside flattening and subflattening methods in Sect. 4 following the process suggested by Holland et al. (2012) and direct the reader to the two aforementioned papers for additional background information on squangles and how they may be used to evaluate quartet splits.

The general philosophy followed here is that, under standard phylogenetic models, evolutionary divergence events are modelled as discrete instantaneous branching of lineages, whereas the subsequent evolution occurs as a continuous time random process of nucleotide substitutions. Thus, if one views the primary difficulty of phylogenetic inference to be the correct identification of branching events (i.e., the inference of the phylogenetic tree), the random nucleotide substitutions that occur on the pendant edges of the tree contribute nothing and make the inference more difficult. Indeed, the above-stated properties of the flattening matrix are contingent on a particular edge being identifiable with evolutionary divergence events occurring some finite time prior. Thus it is valuable to understand the theoretical behaviour of the flattening matrix as time passes under the given Markov model of sequence evolution. The relevant matrix transformation rule is well understood (and was presented in Allman and Rhodes 2008) and is crucial to the theoretical derivation of the rules for the rank of the flattening matrices. Exploring these ideas further, Sumner (2017) showed analogous transformation rules are obtainable from ‘subflattenings’, which are particular submatrices of the flattening matrices. These submatrices have significantly smaller dimensions than the flattenings, reducing the dimension from $k^{|A|}\times k^{|B|}$ to $({|A|}(k-1)+1)\times ({|B|}(k-1)+1)$ for a split *A*|*B*, where *k* is the size of the state space (for example, $k=4$ for the state space of DNA nucleotides $\kappa := \{A,C,G,T\}$). Thus the subflattening has the advantage of having dimensions which are linear in the size of each split, and Sumner showed that the rank conditions on the subflattenings matrices can also be used to (theoretically) identify splits corresponding to edges in the true phylogenetic tree (Sumner 2017).

The purpose of the present work is to show the relative performance of these methods—in terms of their ability to identify edges in the true tree—under a simple simulation framework. In particular, while the theoretical properties of the subflattening hold under the general Markov model, we focus on simulating under the Jukes–Cantor model with varying sequence length and branch lengths. Throughout, ‘branch length’ refers to the expected number of substitutions per site. While we expect that the theoretical properties of the subflattening will hold under the coalescent, we do not consider such complications here, in order to keep the presentation and comparisons as clean and clear as possible.

In Sect. 2, we provide the relevant background definitions and theorems, including the flattening and subflattening rank theorems. In Sect. 3, we identify some important considerations in the implementation of flattenings and subflattenings and describe the software we have developed to construct such matrices and identify true splits. In Sect. 4, we detail the simulations we performed in order to evaluate the performance of subflattening matrices in comparison with flattenings and squangles. The results of the simulations are discussed in Sect. 5, and we conclude with a discussion in Sect. 6 including suggestions for future work.

## Background

For a given set of taxa *X*, a *split* is defined as *partition* of *X* into two sets *A* and *B* and is written *A*|*B* (or equivalently *B*|*A*). We may also omit set notation. For example, if $X=\{0,1,2,3\}, A=\{1\}$ and $B=\{0,2,3\}$, then the split *A*|*B* can be written 023|1. Since splits are bipartitions, we can also omit one side of the split when *X* is clear. In this case, we might write 023|1 as 023 or simply 1. We define the *size* of a split as the size of the smallest subset (for example, the size of 023|1 is 1) and note that the size of a split can be thought of as a measure of its *balance*; this is because for a fixed number of taxa, the larger the size of a split, the more balanced the taxa are across the two subsets. Splits are a natural idea in phylogenetics, because each edge in a phylogenetic tree can be identified with a split. To see this, notice that removing an edge from a tree creates two smaller trees and hence a partition of the taxa into two sets; the induced partition can be represented as a split. Of course, not all splits will represent edges in a given tree. For example, if a tree displays two taxa as a cherry, then any split with size more than 1 which separates these two taxa will not correspond to any edge in the tree. Splits with size equal to 1 will always correspond to leaf edges, and for this reason they are referred to as *trivial* splits. Given a tree, we say that splits which correspond to edges are *displayed* by the tree and refer to them as *true* splits. For example, trivial splits are always true splits. Splits which are not displayed by the tree are referred to as false splits. Splits are *compatible* if there exists some tree which display both splits. For example, the splits 01|23 and 02|13 are not compatible on any tree. Due to the correspondence between true splits and edges, the complete set of true splits uniquely defines the tree (Steel 2016, Proposition 2.4).

Fundamentally, inference of phylogenetic trees amounts to the identification of true splits. Importantly, there are far fewer splits on *t* taxa than there are trees on *t* taxa. The number of splits is given by $2^{(t-1)}-1$ whereas the number of unrooted binary trees is given by $(2t-5)!! = \frac{(2 (t-2)) !}{2^{(t-2)} (t-2) !}$ (which grows like $2^t t!$). It is also worth noting that a tree without branch lengths (or with branch lengths which are all equal) may have a number of splits which are symmetrically equivalent (that is, they are invariant under an action of the automorphism group of the tree under vertex permutations). For example, for the balanced four-taxon unweighted tree with leaf labels $\{0,1,2,3\}$ and cherries $\{0,1\}$ and $\{2,3\}$, we can say that the (false) splits 02|13 and 03|12 are symmetrically equivalent. A true split on a tree induces two sub-trees which are disjoint (that is, they share no edges in the original tree). A false split, however, will always give rise to two sub-trees which share one or more internal edges in the tree. The number of shared edges between the two sub-trees may be taken as a measure of ‘how false’ a given false split is on a tree and is a statistic which explains some of the results displayed in the next section.

Another simplistic measure is the parsimony score for a split. Recall that the parsimony score for a site pattern is the minimum number of state-changes that need to occur on the edges of the tree in order to observe the given pattern at the leaves (Steel 2016, Chapter 5.2). Similarly, the parsimony score for a split *A*|*B* is the parsimony score for a pattern which assigns one state to the taxa in *A* and another state to the taxa in *B*. Of course, both of these measures require knowledge of the true tree.

In practice, we can obtain a measure of how likely it is (in a colloquial sense) for a split to be true, using the rank properties of flattening and subflattening matrices. The flattening rank properties are presented in the following theorem.

### Theorem 2.1

Given a phylogenetic tree *T* and state space $\kappa = \{1,...,k\}$, along with it is associated site-pattern probability distribution *P*, the *flattening* matrix $\textrm{Flat}_{A|B}(P)$ computed from a split *A*|*B* has generic rank $\textrm{rank}(\textrm{Flat}_{A|B}(P)) = k$ if the split *A*|*B* is displayed by *T*, and $\textrm{rank}(\textrm{Flat}_{A|B}(P)) = k^{p_{A|B}}$ otherwise, where $p_{A|B}$ is the parsimony score of the split *A*|*B* on the tree *T*.

### Proof

A proof is provided by Casanellas and Fernández-Sánchez (2011), strengthening a previous result by Eriksson (2005). $\square$

**Fig. 1:**
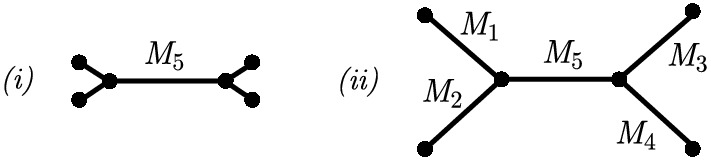
Two quartet trees. The left tree has leaf edges with branch lengths of zero (depicted by short edges), and in the right tree these branches are extended

Note that by ‘generic rank’ we simply mean the rank of the flattening assuming the probability distribution *P* arose from non-singular Markov matrices.

To provide some intuition behind the flattening construction and associated rank property, we introduce an example. Consider the simple phylogenetic tree shown in Fig. 1 (*i*), where the leaf edges have zero branch length, which corresponds to Markov matrices equal to the identity matrix. We refer to the site-pattern probability distribution for this tree as $P_0$. Below are the flattenings for the two splits 01|23 and 02|34 on this tree, where the omitted entries are zero. Since a flattening is simply a configuration of site-pattern probabilities, we see a large number of zero entries, corresponding to the patterns which cannot occur due to the leaf branches having zero edge length.In this simple case, we can see that the rank properties for true and false splits hold. That is, with $k=2$, the rank of the 01|23 flattening is $2=k$ and the rank of the 02|13 flattening is $4=k^2$. We now consider growing the leaf edges to produce the tree shown in Fig. 1 (ii). In terms of the site-pattern probability distribution, assigning the matrices $M_1, M_2, M_3$ and $M_4$ to the leaves of the tree is achieved via the transformation,

$$\begin{aligned} P = M_1 \otimes M_2 \otimes M_3 \otimes M_4 \cdot P_0, \end{aligned}$$

where $\otimes$ is the standard Kronecker product for matrices, as shown in Sumner et al. (2008a). Note that *P* is the site-pattern probability distribution of the tree shown in Fig. 1. Further, the effect on the flattening can be written just as simply. For instance, the flattening for the split 01|23 transforms as,

1 $$\begin{aligned} \textrm{Flat}_{01|23}(P) = (M_1 \otimes M_2 ) \cdot \textrm{Flat}_{01|23}(P_0) \cdot (M_3 \otimes M_4)^T \end{aligned}$$

One can observe that if the matrices $M_i$ are non-singular (which is generically true) the rank is unchanged under this transformation, that is the rank of $\textrm{Flat}_{01|23}(P)$ is (generically) equal to the rank of $\textrm{Flat}_{01|23}(P_0)$.

We will discuss shortly how the rank of flattenings can be evaluated in practice, but first we note that in general, these matrices are exceedingly large. The subflattening is a similar construction which is much smaller in dimension, $(|A|(k-1)+1) (|B|(k-1)+1)$ for a split *A*|*B*, compared to $k^{|A|} \times k^{|B|}$ (the dimension of the flattening) (Sumner 2017). To understand the theoretical origin of subflattenings, we give an illustrative explanation using the simplest possible scenario: binary sequence data ($k=2$) and four taxa ($t = 4$). The general case is presented in Sumner (2017).

For the binary states, one can express a general $2\times 2$ Markov matrix as

$$\begin{aligned} M= \left( \begin{matrix} 1-a &{} a\\ b &{} 1-b \end{matrix} \right) . \end{aligned}$$

Following the well-known Hadamard conjugation (Hendy et al. 1994), we consider what happens to this matrix under conjugation by a Hadamard matrix :Note that the constraint ${a,b}\in [0,1]$ is converted to ${v,z}\in [-1,1]$. Now, multiplying two matrices of the above form gives

$$\begin{aligned} \left( \begin{matrix} 1 &{} v\\ 0 &{} z \end{matrix} \right) \left( \begin{matrix} 1 &{} v'\\ 0 &{} z' \end{matrix} \right) = \left( \begin{matrix} 1 &{} vz'+v'\\ 0 &{} zz' \end{matrix} \right) , \end{aligned}$$

This transformation rule tells us algebraically exactly what is happening to the substitution parameters on a single branch of a phylogenetic tree as time passes.

More generally, if we consider a phylogenetic tree and concentrate on the two lineages and substitutions between the $2^2=4$ pairs of binary states as time passes, algebraically the independence of substitutions on the two lineages can be incorporated by taking the tensor (Kronecker) product of two Markov matrices $M_1\otimes M_2$. Working with the Hadamard conjugated matrices,

$$\begin{aligned} M_1\otimes M_2 \mapsto (HM_1H^{-1})\otimes (HM_2H^{-1})= \left( \begin{matrix} 1 &{} v_1\\ 0 &{} z_1 \end{matrix} \right) \otimes \left( \begin{matrix} 1 &{} v_2\\ 0 &{} z_2 \end{matrix} \right) = \left( \begin{matrix} 1 &{} v_2 &{} v_1 &{} v_1v_2\\ 0 &{} z_2 &{} 0 &{} v_1z_2\\ 0 &{} 0 &{} z_1 &{} z_1v_2\\ 0 &{} 0 &{} 0 &{} z_1z_2 \end{matrix} \right) . \end{aligned}$$

The key idea presented in Sumner (2017) was that all of the substitution parameters are contained in upper-left $3\times 3$ submatrix

$$\begin{aligned} \left( \begin{matrix} 1 &{} v_2 &{} v_1 \\ 0 &{} z_2 &{} 0 \\ 0 &{} 0 &{} z_1 \end{matrix} \right) . \end{aligned}$$

That is, one row and column can be removed without losing information. The Hadamard transformation on Markov matrices can be translated to an equivalent transformation on the site-pattern probability distribution, where the transformed probability distribution is written,

$$\begin{aligned} {\widehat{P}} = (H \otimes H \otimes H \otimes H) \cdot P. \end{aligned}$$

Further, the effect of the transformation on the flattening can be written,

$$\begin{aligned} \textrm{Flat}_{A|B}({\widehat{P}}) = (H \otimes H) \cdot \textrm{Flat}_{A|B}(P) \cdot (H \otimes H)^T. \end{aligned}$$

While we leave the details for the interested reader, one can easily observe the interplay between the Hadamard transformation and the flattening transformation. Returning to our running example, transform the flattening $\textrm{Flat}_{01|23}(P)$ and obtain

$$\begin{aligned}&\textrm{Flat}_{01|23}({\widehat{P}}) \\&\quad = (H\otimes H) \cdot \textrm{Flat}_{01|23}(P) \cdot (H\otimes H)^T \\&\quad = (H\otimes H)(M_1\otimes M_2 ) \cdot \textrm{Flat}_{01|23}(P_0) \cdot (M_3\otimes M_4)^T (H\otimes H)^T\\&\quad = \ (H\otimes H) (M_1\otimes M_2 ) (H\otimes H)^{-1} \cdot \textrm{Flat}_{01|23}(\widehat{P_0}) \cdot ((H\otimes H)^T)^{-1} (M_3\otimes M_4)^T (H\otimes H)^T \\&\quad = (HM_1H^{-1})\otimes (HM_2H^{-1}) \cdot \textrm{Flat}_{01|23}(\widehat{P_0}) \cdot ((HM_3H^{-1})\otimes (HM_4H^{-1}))^T, \end{aligned}$$

which is equivalent to Eq. (1) after transforming the flattening and Markov matrices on the right-hand side.

The previously mentioned redundant rows and columns mean that we can take a sub-matrix of the transformed flattening to obtain the subflattening, and all of the relevant information is maintained. We direct the reader to Sumner (2017) for details on selecting the appropriate sub-matrix to extract in the general case.

As a simple example, the subflattening *S* obtained by extracting the relevant $3\times 3$ matrix from $\textrm{Flat}_{01|23}(\widehat{P_0})$ is,

$$\begin{aligned} S = \begin{bmatrix} \small p_{0000} + p_{0011} + p_{1100} + p_{1111} &{} p_{0000} - p_{0011} + p_{1100} - p_{1111} &{} p_{0000} - p_{0011} + p_{1100} - p_{1111} \\ p_{0000} + p_{0011} - p_{1100} - p_{1111} &{} p_{0000} - p_{0011} - p_{1100} + p_{1111} &{} p_{0000} - p_{0011} - p_{1100} + p_{1111} \\ p_{0000} + p_{0011} - p_{1100} - p_{1111} &{} p_{0000} - p_{0011} - p_{1100} + p_{1111} &{} p_{0000} - p_{0011} - p_{1100} + p_{1111} \end{bmatrix}. \end{aligned}$$

One can quickly observe that the second and third rows are identical, and so the subflattening matrix *S* is rank-deficient, just as the associated flattening $\textrm{Flat}_{01|23}(\widehat{P_0})$ was. In general, subflattenings have a rank property analogous to Theorem 2.1.

### Theorem 2.2

Let *T* be a tree with site pattern probability distribution *P*. Let *A*|*B* be a split. A subflattening matrix $\textrm{Subflat}_{A|B}(P)$ has generic rank *k* if *A*|*B* is displayed by *T*, and rank $(k-1)p_{A|B}+1$ otherwise, where $p_{A|B}$ is the parsimony score of the split *A*|*B*.

### Proof

See Sumner (2017). $$\square $$

Note that for the remainder of this paper, we will assume that $k=4$, referring to the size of our state-space of DNA nucleotides $\kappa :=\{A,C,G,T\}$.

As stated above, the dimensions of subflattenings are greatly reduced in comparison with the flattenings (exponential to linear). However, this comes at the price of the Hadamard transformation which means that the entries of the subflattenings are not directly obtainable from counting patterns in the sequence alignment. That is, entries do not correspond to single site-patterns, as they do for flattenings. Despite this, the subflattenings can still be computed efficiently, as we describe in Sect. 3.

After flattenings and subflattenings have been constructed using observed pattern frequencies from a sequence alignment, extracting information from them involves evaluating how ‘close’ the given matrix is to having rank *k*. Singular value decomposition has often been suggested in the literature as a way to evaluate this (Allman et al. 2017; Eriksson 2005), due to the connection between singular values and low-rank matrix approximation (Eckart and Young 1936). Put briefly, the sum of all but the *m* largest squared singular values of a matrix *F* is the distance between *F* and the closest rank-*m* under the Frobenius norm. We are interested in the case where *m* is set to the number of states, *k*, as in the flattening and subflattening rank theorems Theorems 2.1 and 2.2. With this connection in mind, Allman et al. (2017) define the ‘split score’:

### Definition 2.3

For flattening (or subflattening) matrix $F_{A|B}$ arising from a split *A*|*B* and a sequence alignment with *k* possible states, the *split score* is

$$\begin{aligned} \displaystyle \textrm{SplitScore}\left( F_{A|B}\right) =\left( 1-\frac{\sum _{i=1}^{k} \sigma _{i}^{2}}{\Vert F_{A|B}\Vert ^{2}}\right) ^{\frac{1}{2}}, \end{aligned}$$

where $\sigma _{1} \ge \sigma _{2} \ge \cdots \ge \sigma _{\min (m, n)} \ge 0$ are the singular values of $F_{A|B}$, and $\Vert F_{A|B}\Vert$ is the Frobenius norm of $F_{A|B}$.

The split score is a normalised measure of the distance to the nearest rank-*k* matrix and requires only the largest *k* singular values to be computed. This saves time on computation. The code we provide to evaluate splits using flattenings and subflattenings utilises this definition of the split score. We note that the split score is simply one approach for extracting information from flattening (or subflattening) matrices, and alternate scores are now being explored; for example, Casanellas et al. (2021) define the semi-algebraic quartet reconstruction (SAQ) method which considers the closest positive semidefinite matrix to each of the three quartet flattenings. More work is needed to evaluate the most appropriate score in practice, particularly for subflattenings.

## Code and Computational Considerations

### Code

Two separate programs were developed for constructing subflattenings, computing split scores and performing the simulations and analysis detailed in Sect. 4. The first implementation, Flatbush was written in C++ by Charleston (2022). Flatbush is a command-line program that accepts either FASTA or NEXUS format sequence alignment data, currently limited to the nucleotide alphabet of characters, $\kappa = \{A,G,C,T\}$. If the NEXUS format is used then a “FLATBUSH” block may be included, which may include instead of an alignment a set of splits with weights (which would otherwise be calculated from an alignment). Splits may be input either as taxon names or in a compact binary encoding and can be entered explicitly or by opting to do all possible non-trivial splits, subject to the number of taxa not being too large (however note that in principle Flatbush can accept any number of taxa and sequences as input). Input arguments may also include an input tree (such as one inferred using some other method) in simple Newick format, and a set of splits of interest. Alternatively, a tree or set of splits may be provided as command-line arguments. Flatbush is available as open-source (at github.com/mcharleston/Flatbush). Please see the GitHub page material for additional information.

A second implementation, SplitP is a python package developed by Stevenson. As SplitP was written independently of Flatbush, we were able to verify our calculations by comparing outputs of both programs with the same inputs. While SplitP is generally slower than the Flatbush implementation, it still includes the optimisations detailed in the remainder of this section and can also compute and evaluate scores for flattening matrices and squangles. For this reason, we use SplitP for the analyses in Sect. 4. As input, SplitP accepts FASTA formatted sequence alignments or a table of site-pattern counts. Alternatively, SplitP also accepts trees in the Newick format. SplitP can compute flattenings and subflattenings using exact site-pattern probabilities or can generate sequence alignments of a given length, making it useful for simulations detailed in Sect. 4. Any sub-model of the general Markov model is supported, as the user can reassign any Markov matrix to each edge in the tree. SplitP is available on PyPI as well as on GitHub (at github.com/js51/SplitP) as open source software.

### Sparsity

Naïve approaches to problems involving very large matrices quickly lead to memory problems. For example, with just 16 taxa, the number of entries in a flattening matrix is $4^{16} \approx$ 4.2 billion. Assuming the entries are 4 bytes each, this leads to over 17 gigabytes of RAM required just to store the flattening in memory. Adding just one more taxon increases this number to more than 4.3 terabytes. Fortunately, flattening matrices are sparse. That is, the number of nonzero entries is typically in the order of the number of rows or columns. There exist a number of computational techniques for dealing with large sparse matrices, including techniques for computing the singular value decomposition, for example. These techniques are implemented in most major scientific computing packages, for example SciPy (Virtanen et al. 2020) for Python or Eigen (Guennebaud et al. 2010) for C++.

*Subflattenings* on the other hand are never sparse, but have dimensions which are quadratic in the number of taxa rather than exponential, and can therefore offer an even greater benefit than sparse matrix methods in certain situations. In a ‘worst case’ scenario, in which each of the $4^t$ site patterns is equally likely to be observed, the expected number of nonzero entries in a flattening matrix is $4^t(1-(1-4^{-t})^L)$, where *L* is the sequence length. In comparison, the expected number of nonzero entries in the subflattening is $(3t+1)^2$, where *t* is the number of taxa. We can therefore determine under this worst-case scenario, the sequence length required to benefit (in terms of memory use) from subflattenings for a given number of taxa. For example, given a 6-taxon tree, a sequence length of around 380 is required, whereas for a 15-taxon tree, a sequence length of over  is required in order to derive benefit from subflattenings. Of course, site-pattern probability distributions are never uniform, and so in practice these thresholds are much higher.

### The Singular Value Decomposition

Due to the ubiquity of the singular value decomposition in numerical methods, packages for computing singular values are heavily optimised. This is true even for the sparse matrix SVD packages, which Allman et al. (2017) employ in order to compute split scores for flattenings. SplitP utilises SciPy’s sparse matrix methods to compute singular values for flattenings and standard methods for subflattenings. Flatbush utilises the C++ package Eigen (Guennebaud et al. 2010). Importantly, the definition of the split score means that only the *k* largest singular values are needed, leading to significant performance improvements for both methods.

### Subflattening Transformation

The bulk of the computation time for constructing the subflattenings from site pattern counts comes from the application of the following transformation of the (theoretical or observed) site-pattern distribution *P* on the set of taxa *X*, with $|X|=t$, as described in Sect. 2.

$$\begin{aligned} P \rightarrow \underbrace{H \otimes H \otimes \ldots \otimes H}_{t \text { times}} \cdot P. \end{aligned}$$

Specifically, the entries of the subflattening all come as sums of site-pattern probabilities,

$$\begin{aligned} \sum _{j_{1}, j_{2} \ldots , j_{t} \in \kappa } H_{i_{1} j_{1}} H_{i_{2} j_{2}} \ldots H_{i_{t} j_{t}} p_{j_{1} j_{2} \ldots j_{t}}, \end{aligned}$$

as presented by Sumner (2017). Fortunately, the products of *H* matrix entries in the above expression can be stored in a dictionary and reused when computing subflattenings for other splits, and even for other sequence alignments. This technique, known as ‘memoisation’, leads to a significant speed improvement, and allows us to perform the simulations we describe in Sect. 4.

### Number of Splits

As we have discussed, sparse matrix methods and the reduced size of subflattenings help to overcome memory constraints for computing split scores. In terms of time constraints, the sequence length is of little concern, as the time taken to construct a subflattening matrix, for example, increases only linearly as the sequence length increases. The real computational challenge which limits the usefulness of rank-based methods as a tool for inference is the number of splits, given by $2^{(t-1)}-1$, where *t* is the number of taxa. A naïve way to infer a tree using the flattening or subflattening split scores is to compute a split score for every possible split, order them from smallest to largest and select compatible splits from the top of the list until the tree is complete. There are of course other methods to obtain a tree by constructing fewer matrices and computing fewer singular values. An example of one such algorithm is presented by Eriksson (2005). Eriksson ’s SVD algorithm requires the computation of only $(t-1)^2 - 3$ split scores to produce an unrooted tree, and builds the tree from the bottom-up, beginning by selecting cherries. In practice, Eriksson ’s SVD algorithm performs poorly (Allman et al. 2017). A possible explanation is that the algorithm compares split scores for splits of different sizes, which are known to be incomparable due to the bias of the flattening split score towards less balanced splits (Allman et al. 2017). While to our knowledge, no other methods for inferring trees on more than 4 leaves using only flattening rank properties have been proposed, it is easy to see how other algorithms can be adapted to use the split scores, for example quartet methods (Grunewald et al. 2006; Ranwez and Gascuel 2001; Reaz et al. 2014; Snir and Rao 2012; Willson 1999). Such methods may also avoid the split size bias. Example of this include work by Fernández-Sánchez and Casanellas (2015), and the software SVDQuartets, which was developed by Chifman and Kubatko (2014, 2015) and used to reconstruct trees based on the split scores for samples of possible quartets under the coalescent model.

## Analysis

This section outlines the analyses that were carried out in order to begin investigating the effectiveness of subflattenings in a few simple scenarios. Analyses comprise mainly of simulations on trees under the Jukes–Cantor model with a selection of branch lengths. The use of such a simple model reduces the number of variables and allows us to survey the properties of subflattening split scores, particularly compared to those for flattenings, as were investigated by Allman et al. (2017) who also focused on the Jukes–Cantor model in their analyses. We note that similar results can likely be obtained under other substitution models, but further investigation is needed as discussed in Sect. 6. The software package used to perform simulations as well as the sliding window analysis performed here is discussed in Sect. 3.

### Distribution of Split Scores on a 6-Taxon Tree

**Fig. 2:**
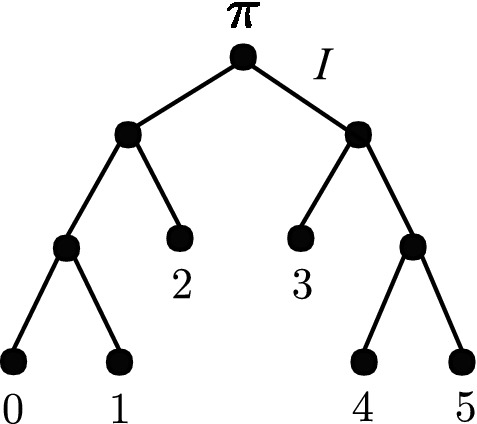
The 6 taxon tree used to simulate site-pattern probabilities and compute split scores for Fig. 6. The branch marked with the identity matrix has length zero, and all nonzero branch lengths are 0.1. The root distribution $\pi$ is equal to $\left[ \tfrac{1}{4},\tfrac{1}{4},\tfrac{1}{4},\tfrac{1}{4}\right]$

We simulated site-pattern frequencies on the balanced six-taxon tree shown in Fig. 2 under the Jukes–Cantor model. For the first simulation, we set all branch lengths equal to 0.1. We first computed exact site-pattern probabilities and then drew 1000 samples from the appropriate multinomial distribution (equivalent to simulating 1000 sequence alignments and counting site-patterns). Split scores were then computed from these empirical site pattern probability distributions. Since all the branch lengths are the same, we can avoid computing split-scores unnecessarily by only computing scores for 9 inequivalent splits. That is, the chosen splits were representatives from the 9 orbits of the action of the tree automorphism group on the set of all splits. The automorphism group consists of all permutations of the leaf nodes which leave the tree unchanged. For example, on a quartet tree with cherries (0,1) and (2,3), the automorphism group is isomorphic to the dihedral group ${\mathcal {D}}_4$, consisting of permutations which swap nodes within each cherry, (01) and (23), the permutations which swaps the two cherries (02)(13) and (03)(12), as well as products of these. The interested reader can verify that the balanced 6-taxon tree shown in Fig. 2 also has automorphism group ${\mathcal {D}}_4$, and the orbits of the group action are as follows:

$$\begin{aligned}&[012|345] = \{012|345\},\\&[01|2345] = \{01|2345,\ 0123|45\},\\&[014|235] = \{014|235,\ 015|234,\ 045|123,\ 023|145\},\\&[024|135] = \{024|135,\ 025|134,\ 034|125,\ 035|124\},\\&[02|1345] = \{02|1345,\ 0345|12,\ 0125|34,\ 0124|35\},\\&[03|1245] = \{03|1245,\ 0245|13,\ 0134|25,\ 0135|24\},\\&[04|1235] = \{04|1235,\ 05|1234,\ 0235|14,\ 0234|15\},\\&[0145|23] = \{0145|23\},\ \\&[013|245] = \{013|245\}. \end{aligned}$$

As an example, the two splits 014|235 and 015|234 are in the same class, since we can obtain one from the other by swapping the labels of the nodes 4 and 5, and this swap leaves the tree unchanged because these nodes form a cherry and have equal branch lengths.

It is important to keep in mind that while these tree symmetries allow us to simplify the results of our simulation detailed below, they are of course not useful for reducing the number of required split-score calculations when inferring trees as the tree symmetries are unknown. Further, if the simulations described here were repeated with varying branch lengths, splits from the same orbit may not produce the same split scores even with infinite sequence length, and so all split scores would need to be computed in order to obtain a complete picture.

Histograms were generated from split scores computed from simulated sequence lengths of 1000 bp and 10,000 bp for both flattenings and subflattenings. In each case, 1000 trials were conducted. That is, we performed 1000 draws from the multinomial distribution in each case, and for each draw computed split scores for each of the splits shown. The entire simulation was then repeated with the same tree, but with every edge length changed from 0.1 to 0.8, emulating a tree which is much more difficult to infer. The results are displayed and discussed in Sect. 5.1.

### Sliding Window Analysis

The next analysis we conducted was a repeat of the sliding window analysis described in Allman et al. (2017) but for subflattenings. We also repeated the analysis using a third method for quartet reconstruction, the squangles (see work by Sumner and Jarvis and Holland et al. 2012 for background on this particular method). The sliding window analysis computes the best split (out of three possible) according to flattenings, subflattenings and squangles for each window in a four-taxon *Anopheles gambiae* mosquitoes data set (Wen et al. 2016). Following the analysis carried out by Allman et al. (2017), we looked at the 2L chromosome of species *An. gambiae*, *An. coluzzii*, *An. arabiensis*, and *An. christyi*. Sliding windows are 10,000 bp long and at each step slide forwards 1000 bp in the alignment. The complete alignment is over 37 million bp, giving approximately 3700 windows in total, as described by Allman et al. (2017). This allows us to see how similar results were between the three methods across the length of the genome.

### Split Balance Bias Analysis

**Fig. 3:**
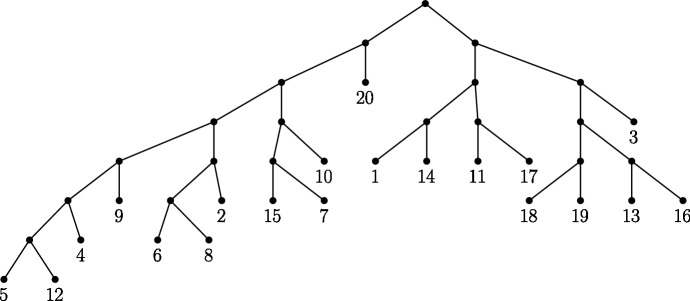
The 20-taxon tree used for split balance analysis described in Sect. 4.3 and in Allman et al. (2017)

In their paper, Allman et al. discuss the bias of flattenings towards less ‘balanced’ splits. Here more ‘balanced’ splits are splits *A*|*B* where |*A*| is closer to |*B*|. For brevity, we refer to splits of size *m* as *m*-splits. According to Allman et al. (2017), this bias is due to the dimension of the space of all matrices of a given shape compared to the subset of these matrices with rank *k*. Due to this bias, Allman et al. (2017) recommend to the reader that splits of different balance not be compared by means of the split score. We hypothesised that subflattenings may exhibit this bias also, but to a lesser extent due to their reduced dimensions. To evaluate this, we evaluated a random selection of 190 *m*-split scores for each size *m* on the 20 taxon tree shown in Fig. 3 under the Jukes–Cantor model with all branch lengths set to 0.05. The number ${20 \atopwithdelims ()2} = 190$ was chosen simply because this is the number of 2-splits (the smallest set when grouping splits by size). This is the same tree used by Allman et al. (2017) to show the existence of split-balance bias in flattening matrices. If the subflattenings are indeed less impacted by split-balance bias, we would expect the average split score to increase less dramatically for increasing split balance, when compared to split scores for flattenings.

### Long Branch Attraction Bias Analysis

**Fig. 4:**
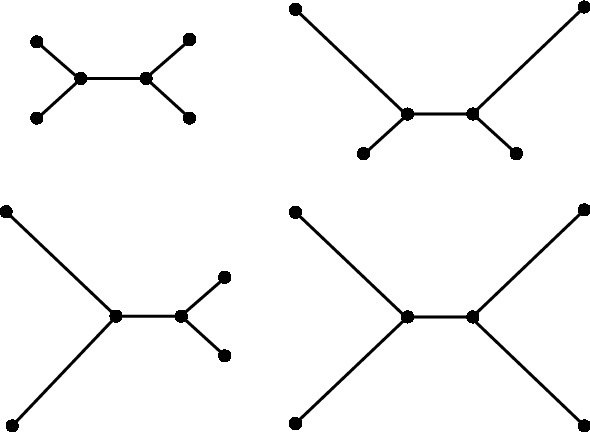
Quartet trees used for the simulations performed in Sect. 4.4. Branch lengths are not to scale

The next set of simulations we performed were designed to give an indication of how susceptible each method is to *long branch attraction*: an important bias affecting some tree construction methods, first described by Felsenstein (1978). The first of these simulations were on quartet trees under the Jukes–Cantor model with short and long branches (see Fig. 4). The short and long branch lengths chosen were 0.05 and 0.5, respectively. The simulation was then repeated with short and long branch lengths of 0.1 and 1.0 (maintaining the ratio of short to long branch length). In both cases, the internal branch was assigned the shorter branch length. We computed site pattern probabilities on each of these trees and drew from the corresponding multinomial distributions, simulating sequence lengths in intervals of 100 bp from 100 bp to 1000 bp. In each case, we performed 100 iterations and determined the number of times flattenings, subflattenings and squangles chose the true split.

We also performed an additional simulation on a *star* tree—that is, a quartet tree with an internal branch length of zero—with two long branches and two short branches. A method which is completely unbiased to long branch attraction would theoretically choose each of the three possible splits a third of the time. A biased method would choose the split which pairs the two long edges more or less often than the other two splits. Finally, we performed a similar simulation, but instead fixed the sequence length at 1000 bp and allowed the long branch lengths to vary from 0.1 to 1.0 substitutions per site with the two other branch lengths fixed at 0.1. The results of these simulations are provided in Sect. 5.4.

## Results

### Distribution of Split Scores on a 6-Taxon Tree

**Fig. 5:**
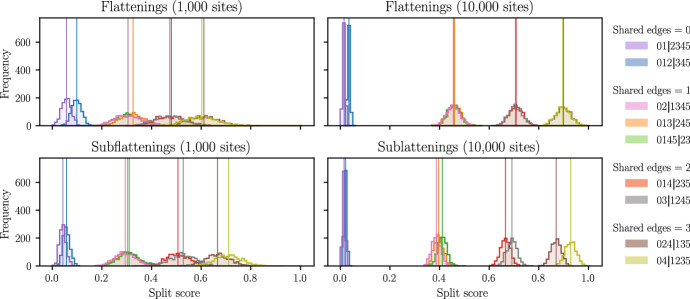
Distribution of split scores for flattenings and subflattenings on simulated pattern frequencies on the 6-taxon tree shown in Fig. 2. Branch lengths are all set to 0.1. The distributions are clearly separated by the number of shared edges

**Fig. 6:**
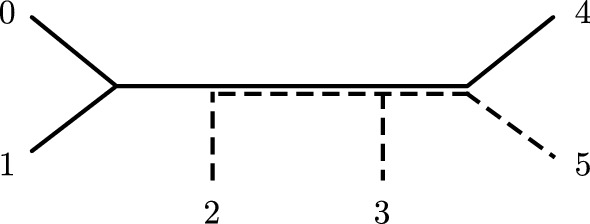
Shared edges example for the split 014|235

The results of our initial simulations are shown in Fig. 5. We saw that split score distributions appeared to be similar between flattenings and subflattenings and were affected similarly by increasing the sequence length. Split scores appeared to fall into four distinct groups, which we know are not a result of tree symmetries due to the chosen splits all being symmetrically distinct. Despite evidence that split scores are influenced by the split size/balance (Allman et al. 2017), we did not see split scores grouped by the split size, rather, split scores appeared to be grouped by the number of *shared edges* for the split on the tree. That is, the number of edges in the tree that are shared between the two subtrees induced by the two subsets of taxa. For example, the split 014|235 on the unrooted 6-taxon caterpillar tree induces the following two subtrees (represented by the solid and dashed lines), which share two edges (Fig. 6).

In our simulations, we commonly saw that the number of shared edges between subtrees induced by a split appeared to explain the ordering and grouping of split scores. In fact, despite the connection between the flattening/subflattening rank and the parsimony score for a split, the number of shared edges is a much better predictor of the split score than the parsimony score for the split. As a simple test, we took the tree in Fig. 2 and drew each edge length uniformly between 0.1 and 1 (Jukes–Cantor) and computed exact site-pattern probabilities and split scores for subflattenings corresponding to each split. Repeating this 20 times and compiling the scores, we fitted a linear model to predict split score based on shared edges, parsimony score and split balance. The best model (according to adjusted $R^2$) used only the number of shared edges, and explained approximately $61\%$ of the variability in the split score. This outcome was similar for flattenings, and increasing the lower bound on the edge lengths to 0.5 increased the $R^2$ value to approximately $89\%$. While, like the split parsimony score, the number of shared edges is unhelpful in discovering true splits (since the true tree must be known in order to compute it), the measure may be helpful in determining exactly which properties of the tree most affect the split score and so it should be investigated further.

**Fig. 7:**
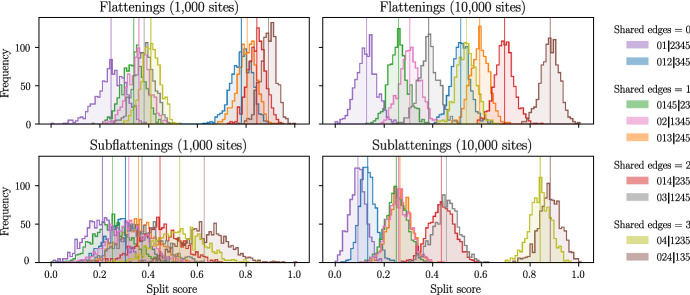
Distribution of split scores for flattenings and subflattenings on simulated pattern frequencies on the 6-taxon tree shown in Fig. 2. Branch lengths are all set to 0.8

For the second simulation, the Jukes–Cantor branch lengths for every branch in the tree were changed from 0.1 to 0.8. This change should make the tree more difficult to infer, since nucleotide substitutions are much more likely to occur along each edge. The results are shown in Fig. 7. We will discuss the flattening scores first. We see that for the more difficult tree and a sequence length of 1000, the flattening split scores are grouped by the split size and then ordered by the number of shared edges. This seems to suggest that split score bias worsens with a weaker phylogenetic signal. That is, when less information about the tree is coming through in the split score, the ordering of the splits becomes more impacted by the size of the split itself, rather than the number of shared edges. Increasing the sequence length to 10,000, we see that the effect of split size has a lesser impact on the distribution of scores; however, the true split 012|345 of size 3 still produced scores which were higher than three false splits of size 2. The subflattenings with a sequence length of 1000 bp seemed to perform similarly to the flattenings with sequence length 10,000 bp, but with scores packed more closely together. Increasing the sequence length to 10,000, the subflattenings produced scores which looked much more similar to the results from Fig. 6, with splits grouped by the number of shared edges, and minimal impact of split size bias. Note that upon increasing the branch lengths from 0.1 to 0.8, the order of the subflattening split scores for the splits 024|135 and 04|1235 reverses; the reason for this is currently unknown. Repeating this simulation with larger trees and varying or randomised branch lengths would be an interesting extension to this work, and while computing the above histograms may be infeasible for all splits on larger trees, splits could be selectively chosen to test the hypothesis that the ‘shared edges’ measure has a large impact on the split score more generally. We leave these investigations for future work.

**Fig. 8:**
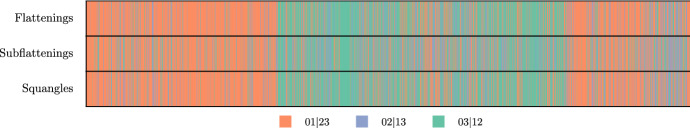
Results of the sliding window analysis of the species (0) *An. gambiae*, (1) *An. coluzzii*, (2) *An. arabiensis*, and (3) *An. christyi* detailed in Sect. 4.2. Each vertical line represents a window, and the colour represents the split which was chosen by each method for that particular window

### Sliding window analysis

**Fig. 9:**
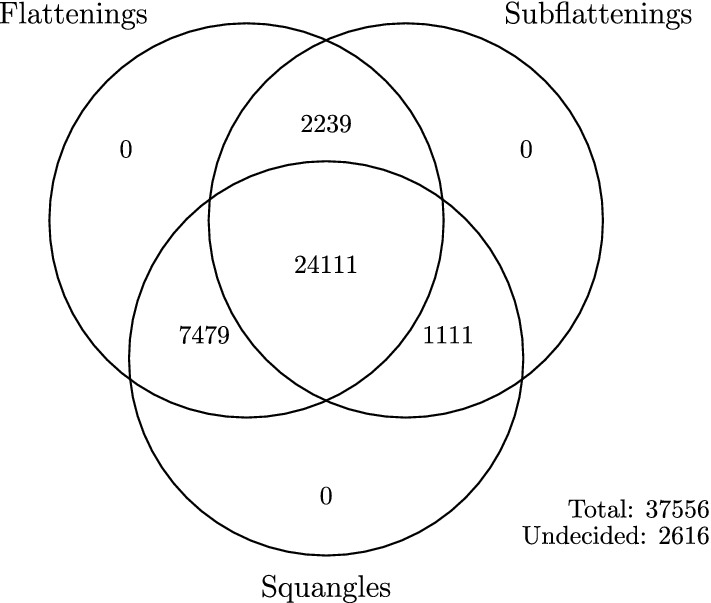
A summary of the results shown in Fig. 8 in the form of a Venn diagram. We can see that the three methods were in agreement for the majority of windows, and of the cases where there was a disagreement between two methods and a third, flattenings and squangles agreed most often

Subflattenings and squangles were able to pick up the chromosomal inversion in the genome, just as flattenings did in the sliding window analysis completed by Allman et al. (2017). The three methods agreed upon the most likely phylogeny for the majority of windows. Interestingly, the most common case involving some disagreement between the three methods was that in which squangles and flattenings chose a different split to the subflattenings. Further, the least common case was when squangles and subflattenings were in agreement with each other, but not with the flattenings. This is visible in Fig. 8 and more clearly in Fig. 9. This was not expected because of the sense that squangles and subflattenings have more in common—they both involve the same transformation of site-pattern probabilities detailed in Sect. 2.

### Split balance bias analysis

**Fig. 10:**
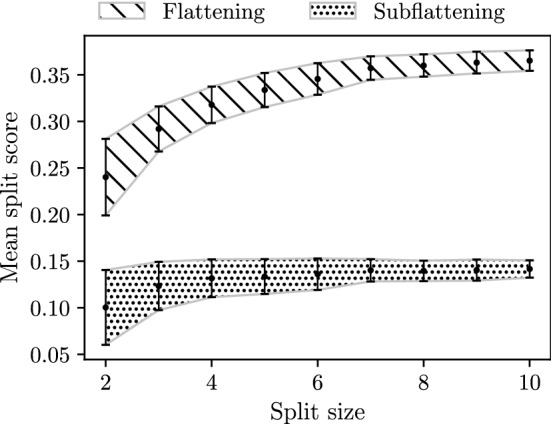
Split scores computed for a sample of 190 splits for each split size on the 20-taxon tree shown in Fig. 3, with branch lengths all set to 0.05, and sequence length set to 500 bp

From our simulations on the 20-taxon tree, both flattenings and subflattenings appeared to be biased towards less balanced splits. Subflattenings appeared to be slightly less biased. Figure 10 shows that, compared to split scores for flattenings, split scores for subflattenings rose more slowly as split size increased. The subflattening scores appear to rise less steeply and flatten off more quickly. The difference is only slight, with the average flattening score increasing by around 52% from the average size 2 split score to the size 10 split score, compared to a 41% increase for the subflattenings.

While we only simulated a single sequence alignment, repeated simulations yielded results which seemed to be stable. Future investigation into the effect of split balance could include a similar simulation study, but with a systematic variation of sequence length, branch lengths and/or tree topology. While Allman et al. suggest that correction to this bias exists theoretically, they deemed it to be of little use in practice (Allman et al. 2017). In our view, the link between the rank properties of flattening/subflattening matrices and the split parsimony score suggests that a practical correction allowing comparison between splits of different sizes does not exist.

### Long branch attraction bias analysis

**Fig. 11:**
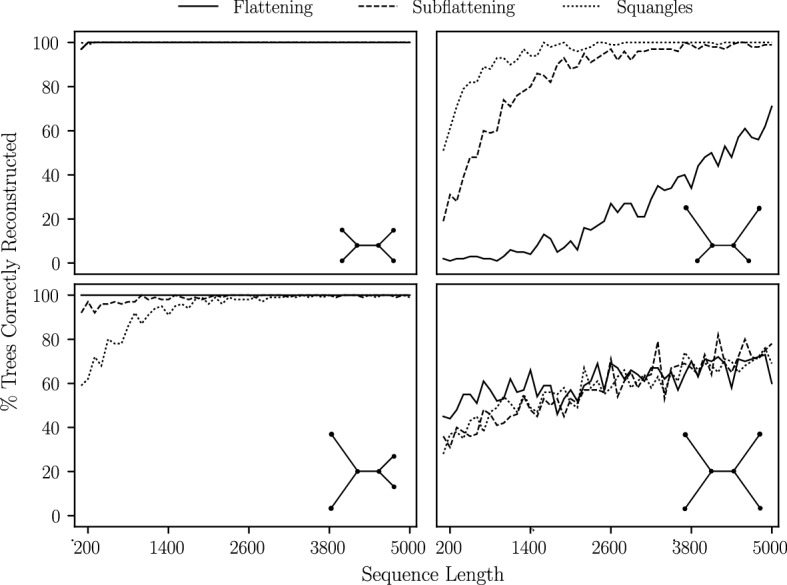
The percentage of times each method was able to choose the correct split from the simulated pattern counts. For each sequence length, we generated 100 different sequence alignments. For each of the four quartet trees, short edges have Jukes–Cantor length 0.05 and long edges have length 0.5

The results of our simulations on quartet trees are shown in Fig. 11. As expected, we observe flattenings, subflattenings and squangles performing similarly on the trees in which all branches were either short or long, with worse over-all performance in latter case. Flattenings showed significantly worse performance on the top-right quartet—an indication that flattenings were incorrectly pairing the long edges together far more often than subflattenings were. Further, flattenings performed slightly better for the bottom left quartet, indicating that the flattenings are more confident in correctly joining the two long branches. These results might indicate that subflattenings and squangles are less impacted by long branch attraction bias when compared to flattenings. Comparing subflattenings to squangles, we saw the squangles correctly reconstruct more trees for the top-right quartet and fewer trees for the bottom-left quartet.

**Fig. 12:**
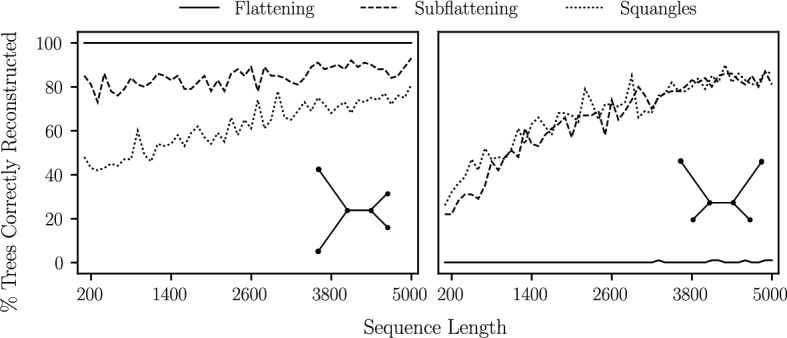
The percentage of times each method was able to choose the correct split from the simulated pattern counts. For each sequence length, we generated 100 different sequence alignments. For each of the four quartet trees, short edges have Jukes–Cantor length 0.1 and long edges have length 1.0

We repeated the simulations on the top-right and bottom-left quartets but with longer branches (short branch length set to 0.1 and long branch length set to 1.0), and the results are shown in Fig. 12. We see that the difference between the flattening and subflattening performance when presented with two long branches was even more pronounced. We note that the long branch length of 1.0 means that substitutions along the long branches are more likely to occur than not, making these trees much more difficult to infer.

**Fig. 13:**
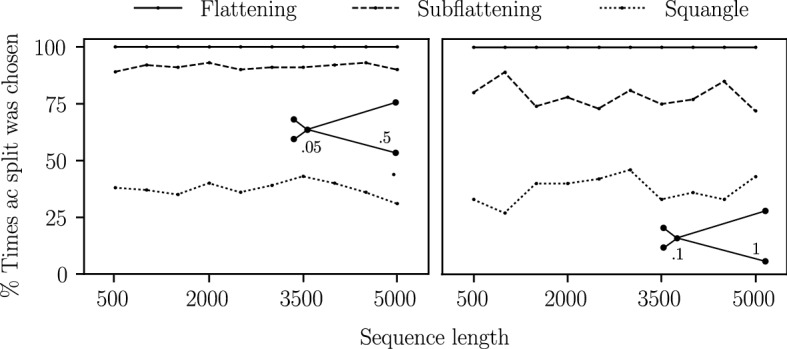
The percentage of times each method chose the split *ac*|*bd* on the star tree on leaves $\{a,b,c,d\}$ under the Jukes–Cantor model with an internal edge length of zero, a branch length of 0.1 for leaves *b* and *d* and longer edges for leaves *a* and *c*. For each sequence length, 100 sequence alignments were generated. Branch lengths are as shown

**Fig. 14:**
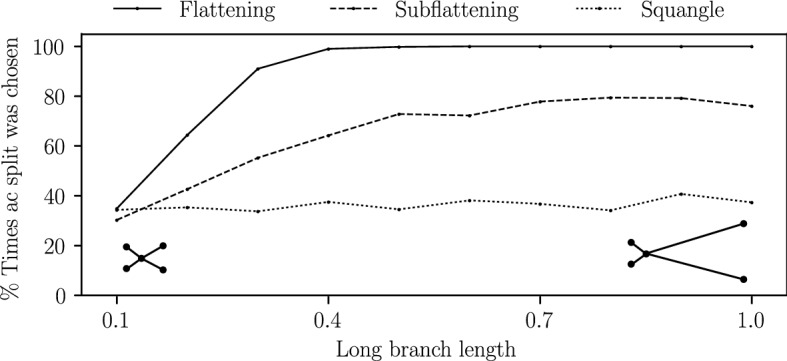
The percentage of times each method chose the split *ac*|*bd* on the star tree on leaves $\{a,b,c,d\}$ under the Jukes–Cantor model with an internal edge length of zero, two short branches of length 0.1 for leaves *b* and *d*, and varying branch lengths for leaves *a* and *c*. For each increase of the long branch length, 100 sequence alignments of length 1000 were generated

Figure 13 shows the percentage of times flattenings and subflattenings correctly reconstructed the star tree with two long branches and an internal branch length of zero. While flattenings paired the two long edges almost every time, subflattenings did so less often. This may further indicate that subflattenings are less biased to long branch attraction, though not completely unbiased, since a totally unbiased method would pair the long edges approximately a third of the time. Increasing all four branch lengths (see the right plot in Fig. 13) seemed to increase the performance of the subflattenings, and Fig. 14 seems to suggest that this effect is due to the increased length of the short branches. Interestingly, the squangles method appeared to be the least affected by long branch attraction bias in these simulations.

While similar results can be obtained under other substitution models, a systematic approach (with varying sequence length, branch lengths, substitution model and model parameters) is needed to explore this further. A theoretical investigation is also recommended in order to confirm and make sense of these observations.

## Discussion

Flattenings and subflattenings, and more generally split and rank based tools, encompass some interesting algebraic and statistical ideas, and motivate methods for phylogenetic inference. While theoretically, subflattenings have analogous rank properties to those of flattenings, there has been no previous investigation into how the two constructions compare in practice. We have presented initial results which show that split scores computed from subflattenings are comparable to those computed using flattenings on real sequence alignments and on alignments simulated under the Jukes–Cantor model on a selection of trees and branch lengths. This analysis gives some support to the use of subflattenings as an alternative tool to flattenings for phylogenetic inference. We also give some evidence that subflattenings may be less impacted by certain biases, namely those arising from split size/balance and long branch attraction. We think this is worth exploring further, but note that some split size bias is likely unavoidable with methods relating to flattenings, due to the relationship between the flattening matrix rank and the parsimony score for the split (see Theorem 2.1), and the fact that the parsimony score is capped by the split size.

It is our hope that this work will contribute to an improved understanding of rank-based methods for constructing phylogenetic trees. There are a number of other avenues available for further research in this area. For example, the analyses presented here could be repeated for additional substitution models (including mixture models), branch lengths and alternate tree topologies with a higher number of taxa. While we have looked at reconstructing quartet trees in this paper, algorithms that utilise flattenings to reconstruct larger trees have been developed (Eriksson 2005) and can instead utilise subflattenings without needing to be adapted in any way. Further, there are numerous methods which reconstruct larger trees using scores for quartets, and flattening or subflattening split scores could be used in these methods to infer trees without constructing larger subflattenings. It would be beneficial to explore tree reconstruction methods that utilise flattenings and subflattenings and systematically compare these to various other approaches in order to determine whether it is practical to incorporate subflattenings into existing tree inference pipelines.

## Data Availability

The *Anopheles gambiae* mosquito DNA sequence alignment data set used for the sliding window analysis in Sect. 4.2 is available at datadryad.org with the identifier 10.5061/dryad.tn47c (Wen et al. 2016)
